# Myopenia and precision (P4) medicine

**DOI:** 10.1002/jcsm.12231

**Published:** 2017-09-24

**Authors:** John E. Morley, Stefan D. Anker

**Affiliations:** ^1^ Division of Geriatric Medicine Saint Louis University School of Medicine 1402 S. Grand Blvd., M238 St. Louis MO 63104 USA; ^2^ Division of Innovative Clinical Trials, Department of Cardiology and Pneumology University Medical Centre Göttingen Robert‐Koch‐Straße 40, D‐37075 Göttingen Germany

**Keywords:** Myopenia, Precision medicine, Muscular dystrophy, Personalized medicine

## Abstract

Precision (P4) medicine represents a new medical paradigm that focuses on Personalized, Predictive, Preventive and Participatory approaches. The P4 paradigm is particularly appropriate for moving the care of persons with myopenia forward. Muscular dystrophies are clearly a set of genetically different diseases where genomics are the basis of diagnosis, and genetic modulation via DNA, oligonucleotides and clustered regularly interspaced short palendronic repeats hold great potential for a cure. The utility of personalized genomics for sarcopenia coupled with utilizing a predictive approach for the diagnosis with early preventive strategies is a key to improving sarcopenic outcomes. The importance of understanding different levels of patient enthusiasm and different responses to exercise should guide the participatory phase of sarcopenic treatment. In the case of cachexia, understanding the effects of the different therapies now available through the P4 approach on muscle wasting is a key to management strategies.

“I am launching a new Precision Medicine Initiative to bring us closer to curing disease like cancer and diabetes – and to give us all access to personalized information we need to keep ourselves and our families healthier.”

~President Obama

State of the Union

January 20, 2015

Myopenia was defined as clinically relevant muscle wasting associated with impairment of muscle function and/or an increase in morbidity and/or mortality.^1^, ^2^ Conditions producing myopenia could be either congenital or acquired. In adults, the two most common causes of myopenia are cachexia^3^ and sarcopenia.^4^

Precision (P4) medicine or patient‐centred medicine is a concept developed by the biologist, Leory Hood.^5^ While he stressed the importance of genomics, metabolimics, transcriptomics and proteomics in developing a personalized profile for each patient to allow more precise care, it also stresses the importance of recognizing the different possible causes of a process in the individual person and the importance of early recognition and prevention of those at risk and the concept that the individual should make their own decision about treatment choices and be actively involved in her own management.^6^ The tenets of this approach can be summarized by the concept of P4 medicine:

Personalized: Utilizing genomics and other molecular diagnostic tools, as well as environmental and lifestyle characteristics to create a personal diagnostic and management plan.
Predictive: Utilize this available information to recognize the risk of an individual developing a specific disease and the likelihood of them responding to different treatments.
Preventive: Based on this knowledge, each individual has their own primary and secondary prevention plans.
Participatory: The data is shared with the individual who then participates in choosing the treatment choices. In theory, this will lead to better compliance.

While the full implementation of P4 medicine is clearly in the future, many of the components are becoming increasingly available and can proactively be introduced at this time. Myopenia represents a set of conditions where rapid uptake of P4 medicine can occur.

## Muscular dystrophy

Muscular dystrophies consist of a variety of genetic disorders resulting in weakening and breakdown of skeletal muscle.^7^ There are nine main categories of muscular dystrophy and over 30 subtypes. These diseases are related to alterations in the structure or function of the dystrophin protein. They are due to mutations in genes having a critical role in muscle function.

Being able to recognize the genetic mutation that causes each of the forms of muscle dystrophy has opened up a variety of methods by which these genes can be manipulated to reverse the disease process. Both *in vitro* and studies in animal models have proven this to be possible. Duchenne muscular dystrophy is due to loss of function in the dystrophin gene. Predominantly, this is due to disruption in the dystrophin protein reading frame.^7^, ^8^ This allows a number of techniques to be developed to provide a precise correction of the dystrophin gene. The length of the dystrophin gene limits the possibility of augmenting the total gene active with cDNAs. For this reason, both viral vectors (adeno‐associated and lentiviral) have been developed to introduce truncated microdystrophin or microutrophin into the DNA.^9^, ^10^ Sleeping beauty transposons represent a nonviral vector approach to insert these microgenes into the genome.^11^ This approach has led to the improvement of dystrophin function to some extent, but not total cure of the disease. Aartsma‐Rus and Krieg^12^, ^13^ developed eteplirsen, an oligonucleotide that interferes with the splicing process allowing the reading frame to be restored. This leads to a partially functional dystrophin. The FDA, based on small clinical trials, approved eteplirsen for the treatment of Duchene muscular dystrophy.

**Figure 1:**
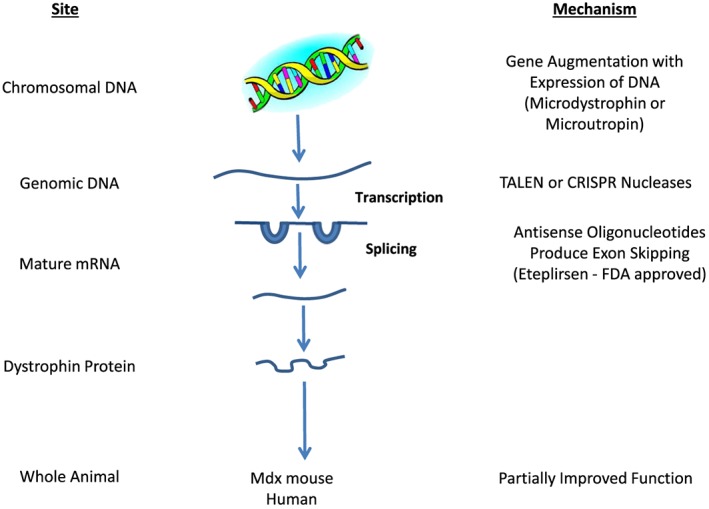
Mechanisms to modulate the gene in muscular dystrophy.

Two programmable nucleases, transcription activator‐like effector nuclease^14^ and clustered regularly interspaced short palendronic repeats (CRISPR),^15^ have been demonstrated to be able to produce gene correction or gene knockout in human stem cells. Adeno‐associated virus‐mediated and RNA guide CRISPR/Cas 9 systems of gene therapy have been developed that produce partially functional dystrophin genes and improve function in the mdx mouse.^16^, ^17^, ^18^ This approach also works in pluripotent human satellite cells.^19^, ^20^ It is important to recognize that CRISPR gene editing also causes unintended mutations. The precision medicine approaches to treating muscular dystrophy are illustrated in Figure 1.^21^

## P4 medicine in sarcopenia

In the Personalized (P1) approach to sarcopenia, a number of allele variations have been identified to be associated with muscle mass and strength.^22^, ^23^, ^24^, ^25^, ^26^ These include myostatin (GDF8, K133R), CNTF and its receptor, vitamin D receptor (VDR Bsml), angiotensin‐converting medicine, androgen receptor gene (CAG repeats), cyclin‐dependent kinase inhibitor 1A, MOD1 and P53 which decreases satellite activation. In addition, small babies predict the presence of low grip strength at 70 years of age.^27^

The Predictive (P2) phase includes a number of screening tests for early sarcopenia that have been developed. For example, Harada *et al.* 28 used sex, age, BMI and adiponectin and sialic acid levels to have a high sensitivity for persons with sarcopenia. In addition, the simple SARC‐F screen (Table 1) has been shown to be highly predictive of persons who are at risk of developing sarcopenia, loss of muscle function, disability and hospitalization. Further, persons with sarcopenia having accelerated loss of muscle when renal cancer is treated with sorafenib is recognized.^29^, ^30^, ^31^, ^32^, ^33^, ^34^, ^35^, ^36^, ^37^, ^38^, ^39^, ^40^ Sorafenib also has an increase in toxicity when given to sarcopenia patient with hepatocellular cancer.^41^

**Table 1:** SARC‐F screen for sarcopenia

Component	Question	Scoring
Strength	How much difficulty do you have in lifting and carrying 10 pounds?	None = 0
		Some = 1
		A lot or unable = 2
Assistance in walking	How much difficulty do you have walking across a room?	None = 0
		Some = 1
		A lot, use aids or unable = 2
Rise from a chair	How much difficulty do you have transferring from a chair or bed?	None = 0
		Some = 1
		A lot or unable without help = 2
Climb stairs	How much difficulty do you have climbing a flight of 10 stairs?	None = 0
		Some = 1
		A lot or unable = 2
Falls	How many times have you fallen in the last year?	None = 0
		One to three falls = 1
		Four or more falls = 2

SARC‐F scale scores range from 0 to 10 (i.e. 0–2 points for each item; 0 = best to 10 = worst) and represent no sarcopenia (0–3) and sarcopenia.^4^, ^5^, ^6^, ^7^, ^8^, ^9^, ^10^

**Table 2:** Patient‐centred precision (P4) medicine applied to sarcopenia

P1: Predictive:	Recognize persons at risk for sarcopenia based on genetic make‐up or being a small baby at birth
P2: Preventive:	Use SARC‐F to screen and then introduce resistance exercise, protein supplementation and vitamin D
P3: Personalized:	Diagnose sarcopenia and identify and manage specific causes, for example, poor blood flow to muscles, low testosterone, cytokine excess, obesity, neuropathic, diabetes mellitus and excess myostatin
P4: Participation:	Recognition and identification of specific exercise approaches based on the individual understanding of the person's muscle type. Work with the person to increase acceptability and understanding of treatment plan and increase compliance

In the Preventive (P3) phase of P4 medicine (Table 2), early recognition that those at increased risk of sarcopenia should lead to advice to increase resistance exercise^42^, ^43^, ^44^, ^45^, ^46^ utilizes leucine‐enriched essential amino acids^47^, ^48^, ^49^ or possibly hydroxymethyl butyrate^50^, ^51^, ^52^, ^53^ and in persons not getting adequate sunlight to provide 1000 IU vitamin D daily.^54^, ^55^, ^56^

This requires that a clear diagnosis of the cause of sarcopenia should be made early in the process. This includes measuring muscle mass and function^57^, ^58^, ^59^, ^60^, ^61^ and a muscle biopsy or measuring C‐agrin to determine whether the person has predominantly neuropathic muscle loss or some other cause.^62^, ^63^, ^64^ Recognition of whether the person has obese sarcopenia represents another part of personalization.^65^, ^66^, ^67^ A decrease in bioavailable testosterone or low dehydroepiandrosterone levels as a cause of muscle loss should be identified.^68^, ^69^, ^70^, ^71^ These persons are more likely to respond to testosterone therapy,^72^, ^73^, ^74^ particularly if they have congestive heart failure.^75^, ^76^, ^77^, ^78^, ^79^, ^80^ Blood flow to muscles is a key cause of muscle loss, especially in diabetes mellitus.^81^, ^82^, ^83^, ^84^ Finally, cytokine excess plays a major role in muscle loss.^85^, ^86^

Animals lacking myostatin have increased muscle mass.^87^ Myostatin binds to the activin II receptors with higher affinity for the IIb affinity.^88^ In older persons, stem cell expression of myostatin is higher than in younger persons.^89^ However, the levels of myostatin in younger and older persons vary considerably in different individuals suggesting that individuals may have their own specific myostatin levels.^90^ LY2495655, a myostatin antibody, increased muscle mass and improved muscle function.^91^ Other studies have shown less dramatic effects of myostatin antibodies in older persons with sarcopenia.^92^ A decoy receptor for activin II receptors increased muscle mass was associated with bleeding.^93^ Persons who have haemorrhagic telangiectasia have an abnormal activin receptor I.^94^ Animal studies suggest that muscle effects are due to the activin II receptor.^95^ In humans with sarcopenia related to femoral fracture, there are increased levels of myostatin and increased phosphorylation of Smad proteins.^96^ These findings suggest that anti‐myostatin antibodies should be preferentially considered in sarcopenic individuals with muscle elevations of the myostatin/Smad pathways, a perfect example of P4 medicine. Preliminary studies in goats and rabbits using CRISPR/Cas9 to knock out myostatin increased muscle mass but had a number of side effects.^97^

The final component of P4 medicine is Participatory. Patients need to be able to choose which therapies they would prefer. An important component of this is to recognize that response to exercise varies enormously in individuals, and 30% of this appears to be related to the person's genetic make‐up.^98^ Churchward *et al.* 99 have suggested that despite this, all persons have some degree of response to resistance exercise. In the end, the person's compliance with the exercise and dietary programs remains the major factor deciding the outcome of the therapeutic program. This was nicely shown in the ‘Look Ahead’ study, where those persons in the upper quartile of time spent exercising a week had significantly better outcomes than those in the lowest quartile.^100^

## Cachexia

Cachexia is a multifactorial syndrome due to a variety of conditions leading to inflammatory muscle mass loss.^101^ While the molecular basis of muscle wasting in cachexia is well established in animals (i.e. cytokines such as TNF, IL‐1, IL‐6, interferon, TNF receptor adaptor protein, associated with the ubiquitin–proteasome system and elevated myostatin), it is less well established in humans.^102^, ^103^ The variability in humans is the result of the multiple different disease causes and genetic variability.

Management of cancer is one of the leading areas in precision medicine. As already noted, muscle function both affects the side effects of cancer chemotherapy, and the therapy can accelerate muscle loss. Immune checkpoint therapy with its release of a cascade of immune systems to attack the cancer is likely to see an acceleration of muscle loss.^104^ For long‐term recovery, it will be important to attempt to protect muscle during these times.

It is recognized that persons admitted to hospital lose as much as a kilogram of muscle in 3 days and that those with sepsis lose even more muscle mass and long‐term muscle function.^105^, ^106^ As part of personalized medicine, it is essential to see that hospitalized patients get adequate amounts of protein (1.5 to 2 g/day) and have resistance exercise to maintain their muscle mass – neither of these approaches are common in hospitals. There is data to support that ICU patients who get out of bed daily have better outcomes.^107^

## Conclusions

P4 medicine represents an explosive new way to approach myopenia. It is clear that the old fashioned approach to medicine that has been disease based will change over the next decade to a personalized medicine where the person's genetic make‐up and other molecular characteristics will determine the approach to the management of disease. To incorporate this into medical practice, we will need to utilize computer‐assisted management. The medicine of today lacks precision in diagnosis and therapeutics—personalized medicine will alter modern medicine placing the emphasis on the patients, their genes, their environment, their response to therapies and their participation in their own care. The myopenias represent an area in which a rapid understanding and deployment of P4 medicine will improve the quality of life of large numbers of persons.

## Ethics statement

The authors certify that they comply with the ethical guidelines for authorship and publishing of the Journal of Cachexia, Sarcopenia and Muscle.^108^

## Conflict of interest

None declared.
